# Behaviour of FITC-Labeled Polyallylamine in Polyelectrolyte Microcapsules

**DOI:** 10.3390/polym15163330

**Published:** 2023-08-08

**Authors:** Alexey V. Dubrovskii, Alexey V. Berezhnov, Aleksandr L. Kim, Sergey A. Tikhonenko

**Affiliations:** 1 Institute of Theoretical and Experimental Biophysics Russian Academy of Science, 3, Institutskaya Str., 142290 Pushchino, Moscow Region, Russiakimerzent@gmail.com (A.L.K.); 2 Institute of Cell Biophysics of the Russian Academy of Sciences, Federal Research Center “Pushchino Scientific Center for Biological Research of the Russian Academy of Sciences”, 142290 Pushchino, Moscow Region, Russia; alexbereg56@gmail.com

**Keywords:** polyelectrolyte microcapsules, polyallylamine, FITC-PAH, 13-layered PMC, PMC, polyelectrolytes

## Abstract

There are many studies devoted to the application of polyelectrolyte microcapsules (PMC) in various fields; however, there are significantly fewer studies devoted to the study of the polyelectrolyte microcapsules themselves. The study examined the mutual arrangement of the polyelectrolytes in 13-layered PMC capsules composed of (PAH/PSS)_6_PAH. The research showed that different layers of the polyelectrolyte microcapsules dissociate equally, as in the case of 13-layered PMC capsules composed of (PAH/PSS)_6_PAH with a well-defined shell, and in the case of 7-layered PMC capsules composed of (PAH/PSS)_3_PAH, where the shell is absent. The study showed that polyallylamine layers labeled with FITC migrate to the periphery of the microcapsule regardless of the number of layers. This is due to an increase in osmotic pressure caused by the rapid flow of ions from the interior of the microcapsule into the surrounding solution. In addition, FITC-polyallylamine has a lower charge density and less interaction with polystyrene sulfonate in the structure of the microcapsule. Meanwhile, the hydrophilicity of FITC-polyallylamine does not change or decreases slightly. The results suggest that this effect promotes the migration of labeled polyallylamine to a more hydrophilic region of the microcapsule, towards its periphery.

## 1. Introduction

Microencapsulation is a technology that enables the protection of sensitive compounds by enclosing them within small spheres, with diameters ranging from 1 micron to several hundred microns. This technology facilitates safe delivery and prolonged functioning of encapsulated compounds. There are currently numerous microencapsulation methods, including spray cooling, extrusion, air flow coating, spray drying, and coacervation, among others [1,2,3,4,5]. The selection of a specific method depends on the properties of the encapsulated substance, as well as the desired morphology and properties of the capsules. However, the methodology for creating polyelectrolyte microcapsules, obtained by the alternate adsorption of polyelectrolytes, is distinguished by its versatility [6].

Polyelectrolyte microcapsules (PMCs) were first obtained in 1998 and, since then, they have been actively studied in the field of polymer nanotechnology [7,8]. PMCs are spherical microcontainers that are prepared using the layer-by-layer (LBL) technique [9]. They are created by alternately adsorbing positively and negatively charged polyelectrolytes onto dispersed nanoparticles and microparticles [7,10,11,12]. The resulting microcapsules have a multilayered structure which offers several benefits over other types of microcapsules. Firstly, the better control over the release of encapsulated materials allows for more precise release of the contents, enabling the microcapsules to be used in a variety of applications [13]. Secondly, the ability to incorporate different types of functional groups allows for the creation of custom microcapsules with unique properties that can serve a variety of purposes [14,15,16,17,18]. Additionally, the multilayered design also provides increased stability to the microcapsules, allowing them to be used in harsher environments where other types of microcapsules may not be as effective [19,20,21]. Overall, the multilayered structure of these microcapsules makes them a versatile and effective tool for a range of applications.

One of the distinctive features of polyelectrolyte microcapsules is their semi-permeability [22]. That is, the PMC shell is permeable to low molecular weight compounds of less than 1 kDa (for example, substrates and products of enzymatic reaction), which allows their use as bioreactors and in diagnostic systems. It is important to note that the semi-permeability of the polyelectrolyte microcapsule shell maintains access to the nutrient medium, allowing for the creation of new types of cell models. For example, in the work of Daria S. Zaitseva-Zotova and her co-authors, the possibility of generating multicellular tumor spheroids (MTS) was demonstrated [23]. It turned out that the proposed model based on multicellular spheroids is more resistant to photodynamic therapy than a two-dimensional monolayer cell culture. Thus, multicellular spheroids can be considered a promising three-dimensional in vitro model for evaluating drug doses or parameters for photodynamic therapy in vitro before conducting preliminary clinical studies. Moreover, polyelectrolyte microcapsules with semipermeable properties are a promising container for various types of cells, while providing access to the nutrient medium. A publication by Amin Vossoughi and Howard W. T. Matthew demonstrates that mesenchymal stem cells can be encapsulated without significant impact on their viability [24]. This approach enables the use of polyelectrolyte microcapsules with cells in bioprinting and other areas of bioengineering.

Another distinctive feature of PMCs is their multifunctionality. Polyelectrolyte microcapsules can be used to solve a wide range of problems due to the encapsulation of various substances, such as inorganic nanoparticles [14,15,16,17,18], carbon nanotubes [25], antibodies [26,27], dyes [28,29,30,31], quantum dots [32,33,34], and others. For example, PMCs can be used in the medical field to target specific cells by encapsulating antibodies that recognize those cells [35]. In the field of electronics, PMCs can be used to encapsulate inorganic nanoparticles or carbon nanotubes, which can then be used to create electronic devices [25,36]. In the field of cosmetics, PMCs can be used to encapsulate dyes, which can then be used to create new colors for makeup products [36,37]. Polyelectrolyte microcapsules containing quantum dots have the potential to create a new generation of optical devices. Galina Nifontova and her co-authors proposed using encapsulated quantum dots to expand their application in obtaining long-term quantitative fluorescent visualization [38].

The next feature is the ability to use polyelectrolytes with different properties when creating the shell of PMCs [39,40]. In particular, the structure of the polyelectrolyte, its molecular weight, and charge density can affect the formation of the PMC shell and encapsulation efficiency [21,41,42]. Additionally, polyelectrolytes can be divided into biodegradable and non-biodegradable. Biodegradable polyelectrolytes, such as polyarginine, polylysine, etc., can be destroyed by proteolytic enzymes to release the encapsulated substance into the incubation medium. Such PMCs can be used in medicine for targeted drug delivery [22,43,44], prolongation of medicine action, and controlled release [16,45,46,47]. For example, De Geest et al. were the first to demonstrate enzymatically triggered capsules with two types of shells, which were actively engulfed by African green monkey kidney cells [48]. The capsules released their loaded substance after enzymatic degradation of the shell. This study showcases the potential of enzymatically triggered capsules to release their content in response to an enzymatic signal and could lead to more efficient and precise drug delivery.

Non-biodegradable polyelectrolytes, such as polystyrene sulfonate and poly-allylamine, are resistant to biodegradation through natural microbial and enzymatic processes. PMCs based on these materials can withstand more rigorous incubation conditions for extended periods of time, making them suitable for use in industrial and medical diagnostics for determining the pH of the medium [22], the concentration of low molecular weight compounds [34,49,50,51,52], and surface charge of metals [53]; in industrial or household water treatment for wastewater purification by sorption [54], and other types of activities. For example, Reshetilov and colleagues conducted an experiment that demonstrated the effectiveness of the immobilization method utilizing encapsulated glucose oxidase in PMC in a diagnostic system [25]. This method retained 75% of the initial analytical signal even after 5 months. Therefore, this method is suitable for diagnostic systems that require long-term stability.

As seen above, many works have been devoted to the application of PMC in various fields. However, there are significantly fewer works studying the polyelectrolyte microcapsules themselves, in particular: the stability of the PMC shell [55,56], its surface charge [53], and ultrastructural organization [57], PMC buffer capacity [58], and the permeability of its shell [59]. Nevertheless, their physicochemical properties, internal structure, and mutual arrangement of polyelectrolytes in the PMC shell are poorly understood, although the study and understanding of these parameters are necessary for successful encapsulation of substances and predicting the effect of various conditions on the structure of the microcapsules themselves, as well as on the encapsulated substances.

For the first time, in the work of Musin et al. [60], the mutual arrangement of polyelectrolytes of the PMC shell of different types was studied: PMCs with a dissolved CaCO_3_ core after preparation, PMCs with an undissolved CaCO_3_ core, and PMCs with encapsulated protein. The results showed that the polyelectrolyte layers were mixed in the PMC shell with a dissolved CaCO_3_ core. However, in this work, 7-layer PMCs of composition (PAH/PSS)_3_PAH were described, while polyelectrolyte microcapsules with a greater number of layers have a different structure and differ in properties such as buffering capacity and electrical conductivity.

**Figure 1:**
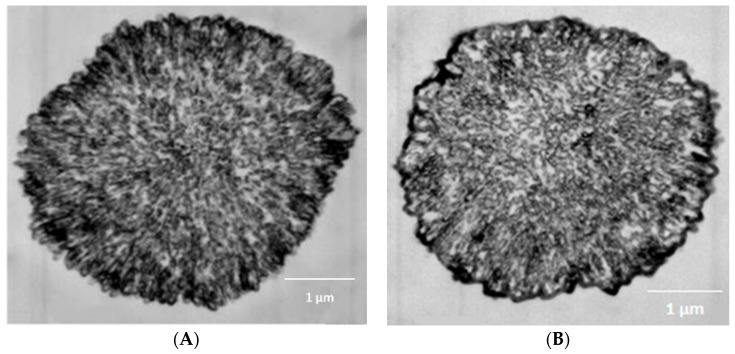
Electron microscopy image of ultrathin sections of polyelectrolyte microcapsules [57]. (**A**) 6 polyelectrolyte layers, (**B**) 10 polyelectrolyte layers.

In particular, in the work of Kazakova et al. [57], photographs of cross-sections of PMCs obtained using electron microscopy showed that polyelectrolyte microcapsules with a dissolved CaCO_3_ core, containing from six to eight polyelectrolyte layers, have no external shell and have a complex internal polyelectrolyte (nanoporous) structure (Figure 1A). And, when the number of layers is nine or more, a brightly pronounced polyelectrolyte shell appears on the surface of the PMC (Figure 1B).

Also, Musin et al. [58] discovered that an increase in the number of layers of polyelectrolyte microcapsules with a dissolved CaCO_3_ core enhances their buffer capacity in a non-linear manner. This implies that the various layers have different levels of protonation depending on their number. Additionally, the number of layers affects the electrical conductivity of the microcapsule shell, as demonstrated in the work of J.B. Schlenoff [61]. As is known, the conductivity of the polyelectrolyte complex depends on the density of ionogenic groups, ionic and non-ionic intra- and intermolecular interactions of individual monomers [62]. These factors can influence the movement of polyelectrolyte layers within the microcapsule shell.

Based on the information above, polyelectrolyte microcapsules with 9 or more layers have a strongly pronounced poly-electrolyte shell compared to 7-layer (PAH/PSS)_3_PAH microcapsules. The shell has a different degree of protonation, density of ionogenic groups, and the number of ionic and non-ionic interactions. These differences can affect the mutual arrangement of polyelectrolytes and their movement within the microcapsule shell when the core is destroyed. Thus, the goal of this study is to study the mutual arrangement of polyelectrolytes in microcapsules with the composition (PAH/PSS)_6_PAH.

## 2. Materials and Methods

Polystyrenesulfonate sodium (PSS) and polyallylamine hydrochloride (PAH) with a molecular mass of 70 kDa Sigma (Merck KGaA, Darmstadt, Germany), fluorescein isothiocyanate (FITC) Sigma (Merck KGaA, Darmstadt, Germany); ethylenediaminetetraacetic acid (EDTA), calcium chloride (CaCl_2_ × 2H_2_O), sodium chloride and sodium carbonate from Reahim (Reahim AO, St. Petersburg, Russian) were used.

### 2.1. Preparation of Fluorescently Labeled PAH

FITC was slowly added to a solution of polyelectrolyte (10 mg/mL) in 50 mM borate buffer, pH 9.0, while stirring the mixture at 300–400 rpm. The FITC and PAH were fused in a molar ratio of 1:100. After that, its solution was incubated for 1.5–2 h. Once the incubation period was over, we dialyzed the solution against water (10 L) overnight, ensuring that there was enough time for the labeled PAH to be purified and ready for use in further experiments.

### 2.2. Preparation of CaCO_3_ Microspherulites

While stirring the 0.33 M Na_2_CO_3_, the 0.33 M CaCl_2_ was added [63]. The stirring time was 30 s. The suspension was maintained until complete precipitation of the formed particles. The process of “ripening” of the microspherolites was controlled with the help of a light microscope. Then, the supernatant was decanted and the precipitate was washed with water and used to prepare PMC. The microparticles were obtained with an average diameter of 4.5 ± 1 μm.

### 2.3. Preparation of Polyelectrolyte Microcapsules

The polyelectrolyte microcapsules were obtained by layer-by-layer adsorbing the negatively or positively charged polyelectrolytes onto CaCO_3_ microspherulites, followed by dissolution of CaCO_3_. At the moment of dissolution of the CaCO_3_ core, the inner space of PMC was filled by interpolyelectrolyte complex [57]. Layer-by-layer adsorption of PAH and PSS on the CaCO_3_ microspherulites surface was carried out in polyelectrolytes solutions (concentration 2 mg/mL + 0.5 M NaCl). After each adsorption, the CaCO_3_ particles with adsorbed polyelectrolytes were triple washed with a 0.5 M NaCl solution, which was necessary to remove unadsorbed polymer molecules. The particles were separated from the supernatant by centrifugation. After applying the required number of layers, the carbonate kernels were dissolved in a 0.2 M EDTA solution for 12 h. The resulting capsules were washed three times with water to remove core decay products. The microcapsules were obtained with an average diameter of 4.5 ± 1 μm. The size, number and ζ-potential of microcapsules was measured using the dynamic light scattering method on a Zetasizer nano ZS device (Malvern, UK). 

### 2.4. Registration of FITC-Labeled PAH Dissociation from Polyelectrolyte Capsules

In order to analyze the dissociation of microcapsules, a fluorescence spectroscopy technique was employed. The microcapsules were composed of several layers, with one layer containing FITC-labeled PAH, which exhibits fluorescence when excited at a wavelength of 525 nm. To begin the analysis, the microcapsules were centrifuged at a rate of 3000 rpm for one minute. After centrifugation, 10 microliters of the supernatant were collected and diluted 40 times to reduce the concentration of the sample. The resulting solution’s fluorescence intensity was then measured. The sample was then shaken, and incubation continued. To record the fluorescence spectra, a Cary Eclipse (Agilent, Santa Clara, USA) instrument was used, which utilized a thermal controlled cuvette with a path length of 1 cm. Excitation of the sample was carried out at a wavelength of 273 nm.

### 2.5. Confocal Microscopy

Polyelectrolyte microcapsules were placed between two coverslips in a drop in PBS medium at a concentration of 2 × 10^6^ particles/mL. Images were acquired with Leica TCS SP5 confocal system (Leica Microsystems, Wetzlar, Germany) as single-image or a Z-stack using HCX PL APO lambda blue 63.0 × 1.40 OIL UV. Image resolution—512 × 512 px, optical resolution—70 nm/px, spatial resolution—140 nm. FITC fluorescence was excited using a 488 nm line of the argon laser (the intensity was set to 4–10% of the maximum). Fluorescence emission was collected at the range of 500–550 nm. The thickness of the optical section in the Z-stack is 0.4–0.9 µM. Images were acquired at 400 Hz scan speed using 3× line averaging to reduce noise. Image processing was performed using FiJi (ImageJ 1.53t) software.

## 3. Results and Discussion

**Figure 2:**
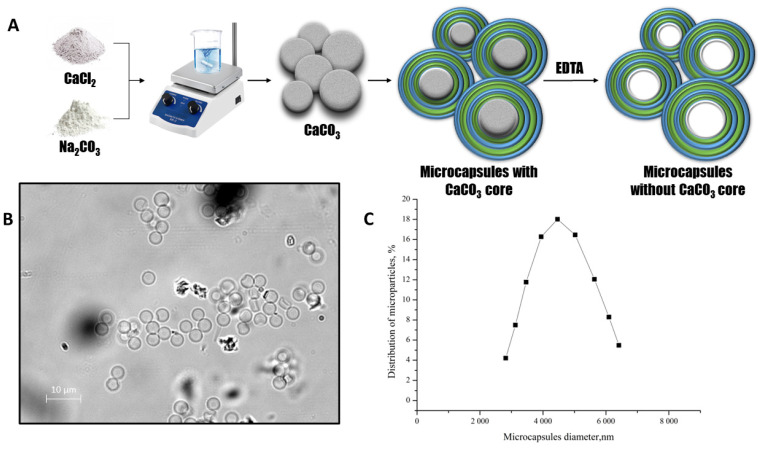
The scheme of the polyelectrolyte microcapsules preparation (**A**). The optical microscopy images of PMC (**B**). The PMC diameter distribution function (**C**).

In this study, our aim was to investigate the mutual arrangement of polyelectrolytes that comprise the structure of polyelectrolyte microcapsules (PMCs). To achieve this, we employed a layer-by-layer adsorption technique using polyelectrolytes polystyrene sulfonate (PSS) and polyallylamine (PAH) onto a CaCO_3_ particle. The particle acted as a template for the formation of the microcapsules. At the final stage of creating polyelectrolyte microcapsules, we dissolved the CaCO_3_ core. The main scheme of the preparation of the polyelectrolyte microcapsules is shown in Figure 2A.

The optical microscopy images of PMC (Figure 2B) demonstrate the morphological homogeneity of microcapsules and the absence of the CaCO_3_ core. The microcapsules had an average diameter of 4.5 μm with an 8.4% polydispersity index (Figure 2C) and a ζ-potential of +20 ± 1 mV.

To investigate the mutual arrangement of polyelectrolytes that comprise the structure of polyelectrolyte microcapsules, we studied the dissociation of each fluorescently labeled polyelectrolyte layer of the microcapsules. We used 13-layered PMCs with a composition of (PAH/PSS)_6_PAH, where PAH acts as the first and last layer in PMC formation. The 7-layered PMCs with a composition of (PAH/PSS)_3_PAH were used as a control based on the study by Musin et al. which showed the effect of mixing polyelectrolyte layers of PMCs (PAH/PSS)_3_PAH after the destruction of CaCO_3_ core [60].

To study the dissociation of a single polyelectrolyte layer of PMC, we used FITC-labeled polyallylamine (PAH) to form a specific positively charged layer of the PMC shell, while non-fluorescently labeled PAH was used to form the rest of the positively charged layers. In the 13-layered PMCs with a composition of (PAH/PSS)_6_PAH, one of the layers was fluorescently labeled: 1st, 3rd, 5th, 7th, 9th, 11th or 13th. In the case of 7-layered PMCs with a composition of (PAH/PSS)_3_PAH, one of the layers was fluorescently labeled: 1st, 3rd, 5th or 7th layer.

**Figure 3:**
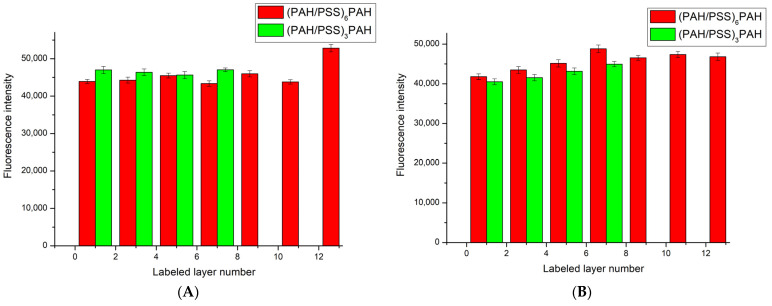
Fluorescence intensity supernatant of PMC, containing different FITC-labeled layers of PAH. (**A**) fluorescence intensity supernatant after 4 h of incubation. (**B**) fluorescence intensity supernatant after 72 h of incubation.

Subsequently, we performed a series of experiments to study the dissociation of the polyelectrolyte microcapsule shell, in which we measured the fluorescence intensity of the supernatant liquid after incubation with microcapsules with a specifically labeled layer. The results obtained are presented in Figure 3.

As shown in Figure 3A, intensity of fluorescence of the supernatant after 4 h of incubation is similar regardless of which layer of the PMC was formed using FITC-labeled polyallylamine. Additionally, the intensity of fluorescence of the supernatant only slightly differs between PMCs with 7 layers and those with 13 layers. Upon further incubation of these PMCs, the intensity of fluorescence changes insignificantly. Therefore, it can be concluded that different layers of PMCs dissociate equally. This is the case for both 7-layer PMCs with the composition (PAH/PSS)_3_PAH and 13-layer PMCs with the composition (PAH/PSS)_6_PAH. However, it is important to note that these polyelectrolyte microcapsules have extremely different morphologies and physicochemical properties. Specifically, a well-defined shell is formed when the number of PMC layers exceeds 9 [57]. Moreover, as the number of layers increases, the buffer capacity [58] and electrical conductivity of the PMCs [61] also change. Therefore, it is proposed to compare the morphology of 7-layer and 13-layer PMCs with the composition (PAH/PSS)_3_PAH and (PAH/PSS)_6_PAH.

**Figure 4:**
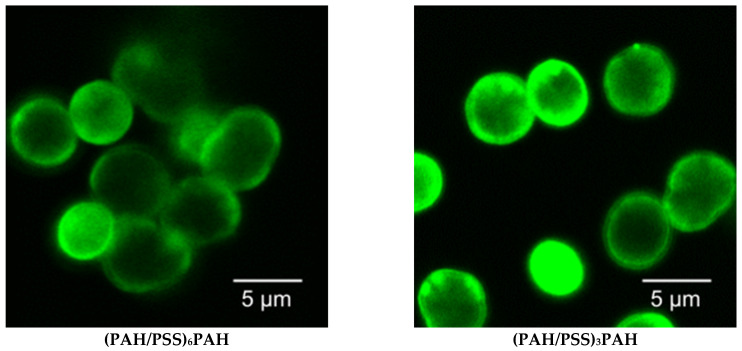
Confocal images of CaCO_3_ microspherolites coated with a polyelectrolyte shell of composition (PAH/PSS)_6_PAH and (PAH/PSS)_3_PAH.

The morphology of PMCs was studied using confocal microscopy. The 7-layer PMCs with composition (PAH/PSS)_3_PAH and 13-layer PMCs with composition (PAH/PSS)_6_PAH, both with fluorescent labelling, were used. Similar to the above experiment, a specific positively charged layer of the PMC shell contained FITC-PAH, while non-fluorescently labeled PAH was used for the remaining positively charged layers. Confocal microphotographs were taken of CaCO_3_ microspherolites covered with a polyelectrolyte shell (13-layer (PAH/PSS)_6_PAH and 7-layer (PAH/PSS)_3_PAH), and the results are presented in Figure 4.

As shown in Figure 4, fluorescence is observed both inside and near the edges of microcapsules containing CaCO_3_ cores. Furthermore, an increase in fluorescence intensity is observed closer to the edges of the microcapsule compared to its internal region. This phenomenon was observed in all microcapsules containing CaCO_3_ cores, regardless of the layer on which FITC-labeled PAH was used during microcapsule formation. This effect may be related to the fact that during the formation of microcapsules, each layer of polyelectrolyte partially adsorbs onto the internal surfaces of CaCO_3_ microspheres [1]. Ultimately, polyelectrolyte layers in microcapsules do not mix or migrate, as confirmed by our earlier studies [60].

**Figure 5:**
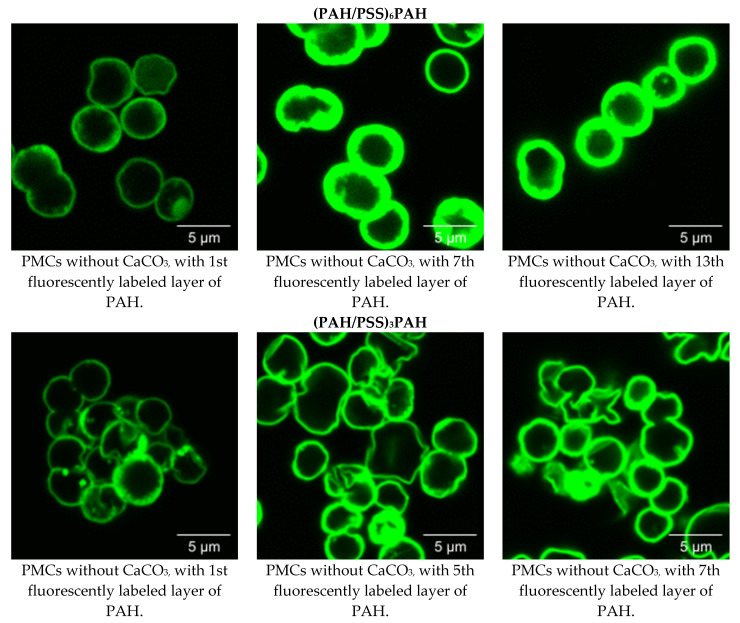
Confocal images of polyelectrolyte microcapsules of composition (PAH/PSS)_6_PAH and (PAH/PSS)_3_PAH.

Subsequently, the morphology of 7- and 13-layer polyelectrolyte microcapsules with removed CaCO_3_ cores and filled with interpolyelectrolyte complexes was studied using confocal microscopy. For this purpose, 7-layer microcapsules of composition (PAH/PSS)_3_PAH and 13-layer microcapsules of composition (PAH/PSS)_6_PAH with fluorescently labeled shells were used. The results are presented in Figure 5.

After removing the CaCO_3_ microspherolites from the PMC, we observed an extremely low intensity of fluorescence in the inner area of the microcapsules (Figure 5). However, we observed a significant increase in fluorescence intensity closer to the edges of the microcapsules. Moreover, this effect is observed regardless of the layer in which FITC-labeled PAH was used during the formation of microcapsules of (PAH/PSS)_3_PAH or (PAH/PSS)_6_PAH composition. Thus, we see that the fluorescently labeled PAH concentrates on the edges of the microcapsules and is almost absent in the inner area.

Taking into account the work of Kazakova et al. (Figure 1), the PMC shell is formed after the adsorption of more than 9 polyelectrolyte layers, and the inner area of the PMC is filled with an interpolyelectrolyte complex already at 6-layered microcapsules [57]. Therefore, in the case of PMC with a composition of (PAH/PSS)_3_PAH, the concentration of fluorescently labeled PAH cannot be associated with the formation of a strongly pronounced shell. At the same time, the low intensity of fluorescence in the inner area of the PMC cannot be associated with the absence of an interpolyelectrolyte complex. Based on the results described above, we can conclude that it is indeed the fluorescently labeled polyallylamine that migrates from the inner area of the PMC to the outer area, closer to the edge of the microcapsules.

Volodkin et al. [9] suggested that during core dissolution, osmotic pressure increases due to the rapid flow of Ca^2+^ and CO_3_^2−^ ions from the nucleus’s inner part to the surrounding solution. This process may have affected the structure and properties of the polyelectrolyte complex formed by the adsorption of polyelectrolyte layers and simplified the movement of fluorescently labeled polyallylamine from the center to the periphery of the microcapsule. A similar effect may result from a decrease in the charge density of polyallylamine after forming a covalent bond with FITC molecules. This may interfere with FITC’s ability to form electrostatic bonds between neighboring amino groups with PSS sulfogroups, resulting in increased hydrophilicity of the interpolyelectrolyte complex PSS—FITC-labeled PAH. As a result, the above-described phenomenon could lead to the migration of fluorescently labeled polyallylamine into the area of the polyelectrolyte microcapsule containing a larger amount of water.

## 4. Conclusions

Research has shown that different layers of polyelectrolyte microcapsules with complex internal polyelectrolyte structure dissociate similarly, whether it is (PAH/PSS)_3_PAH (7-layered) or (PAH/PSS)_6_PAH (13-layered). Despite this, the 13-layered capsules differ from the 7-layered ones by the presence of a formed shell. This result allows us to conclude that regardless of the number of PMC layers, their movement occurs during the dissolution of the CaCO_3_ core.

Additionally, it has been shown that fluorescently labeled polyallylamine migrates from the inner region of the PMC to the outer region closer to the edge of the microcapsule. This effect may be associated with an increase in osmotic pressure caused by the rapid flow of Ca^2+^ and CO_3_^2−^ ions from the inner part of PMC to the surrounding solution during the dissolution of the CaCO_3_ core. In turn, this process could affect the structure and properties of the polyelectrolyte complex that forms during the adsorption of polyelectrolyte layers and facilitate the movement of PMC polyelectrolytes. Moreover, a similar effect may result from a decrease in the charge density of polyallylamine after forming a covalent bond with FITC molecules. This may interfere with FITC’s ability to form electrostatic bonds between neighboring amino groups with PSS sulfogroups, resulting in increased hydrophilicity of the interpolyelectrolyte complex PSS—FITC-labeled PAH. As a result of all of the above, it can be assumed that this effect contributes to the migration of FITC-labeled polyallylamine to a more hydrophilic region of the microcapsule, i.e., to its periphery.

The obtained data can contribute to a better understanding of the migration mechanisms of polyelectrolytes inside microcapsules, which can be important for further research in the field of materials science. In addition, these results may be useful for further development of scientific research in the field of modification of the polyelectrolyte microcapsule shell.

## Data Availability

Not applicable.
